# Medicine

**Published:** 1907-12

**Authors:** J. Michell Clarke


### Progress of the Medical Sciences.

### MEDICINE.

**Course of the fibres of taste.**—With regard to the vexed question as to the course of the taste-fibres, Morriston Davies, in a very valuable paper on the "Functions of the Trigeminal Nerve," based partly on experimental evidence, and partly on the material afforded by fifty cases in which the Gasserian ganglion had been removed,[1] brings forward the following evidence. The difference of opinion is chiefly as to the connection between the chorda tympani and the brain, whether taste impulses pass by the trigeminus or by the facial or glossopharyngeal route. The best evidence dealing with the relationship of the fibres of taste and the trigeminus is derived from the examination of patients after removal of the Gasserian ganglion. The following table gives the result in cases examined more than one month after the operation :—

| Name of examiner. | Total cases. | Taste unaffected. | Taste impaired. | Taste lost. |
|---|---|---|---|---|
| Krause | 5 | 1 | 2 | 2 |
| Cushing | 18 | 17 | 1 | — |
| Davies | 17 | 15 | 1 | 1 |
| | 40 | 33 | 4 | 3 |

Dr. Davies considers that in face of these figures it is impossible any longer to consider the trigeminus as the normal channel through which taste-fibres reach the brain. Explanations of the occasional disturbance, either temporary or permanent, of taste after removal of the Gasserian ganglion have been attributed to (1) injury to the superficial petrosal, which connects the geniculate ganglion with the glossopharyngeal, during the operation or by subsequent formation of scar tissue ; (2) to injury to cells of geniculate ganglion (Dixon) ; or (3) to some interference with chordal (Krause) transmission, brought about by a mechanical or toxic disturbance due to degeneration of the N. lingualis (Cushing).

As to the course of the taste-fibres, the author concludes that clinically evidence has been brought forward which supports those who hold the view that the fibres pass by the pars intermedia, as well as to those who favour the path by the glossopharyngeal, but from morphological, developmental and histological observations, he inclines to the opinion that the gustatory

[1] *Brain*, 1907, xxx. 219.

impulses reaching the geniculate ganglion by the lingual and chorda tympani, pass thence to the brain in the pars intermedia.

**Experimental atheroma.**—In view of the clinical work that has been done in recent years on estimation of the blood pressure, and of the importance of elucidating the causes of atheroma and arterio-sclerosis, the results of experiments made by Dr. G. R. Rickett are of importance.[1] The animals used were rabbits, since by way of the superficial veins of the ear an easy channel for daily intravenous injections is afforded. The drugs employed were adrenalin, squill, barium chloride, tobacco and nicotine.

The experiments show that adrenalin is not the only substance that can cause atheroma in rabbits, and that the cause of the disease, if a toxin, cannot be a toxin peculiar to this substance. The author holds that a mechanical factor, rise of blood pressure, is alone responsible, and that any procedure which raises the blood pressure, whether it be physical or the administration of drugs, provided that the pressure induced be sufficient, will cause atheroma. In applying these results to human pathology, it is to be remembered that these animals have only been experimented with for a few months. In three out of four animals treated with either tobacco or nicotine the author succeeded in producing atheroma; he assumes for the failures that the individuals had a high resistance to the effect of the drug, and remarks that other observers have found it difficult to produce the disease in young animals, and also that the more weakly an animal is the more readily are changes in its vessels produced. He succeeded in producing atheroma with both barium chloride and squill; with the latter, however, the changes are not so severe and extensive as with adrenalin, tobacco, nicotine and barium, but show the same microscopical features. Adrenalin is by far the most certain agent to produce these lesions; this would be expected, if we suppose that atheroma is originated by the damage done by periods of high blood pressure, for adrenalin is by far the most potent agent in this respect.

The author thinks there is no evidence to support the views that the patchy character of the disease is due to the effect of the drug on the vasa vasorum. Further, a mechanical theory affords a reason for the affection of the large trunks only, since it is only in the proximal portion of the vascular system that the pressure rises high enough to effect so great a destruction of elastic tissue. The occurrence of small plaques of atheroma at points of bifurcation, where ascending arteries rise from the artery, is due to the high blood pressure at this early point of the course of these arteries. No changes were found in the

[1] *J. Path. and Bacteriol.*, 1907, xii. 15.

pulmonary artery, because the pulmonary system has no vaso-motor nerves, and is but poorly provided with vessels.

Experiments with potassium cantharidate, chosen because it acts as an irritant but causes no rise in blood pressure, were negative, thus proving that a merely irritant effect cannot cause the disease in question. Without going into detail, it may be said that the earliest changes consist in stretching, frequent breaking and interruption of the elastic fibres, followed later by involvement of the muscle fibres in the degenerative process, and deposition of calcium salts. The nearest parallel in human pathology to the experimental disease is the calcification of the media in the peripheral vessels, and the most important conclusion to be drawn from these experiments is that high blood pressure certainly causes great damage to the arterial system.

**Chloride deprivation in treatment of renal anasarca.**—Prof. Strauss[1] says that chloride deprivation is to be used, not as some have urged for the treatment of diseases of the kidney in general, but for the prevention and treatment of certain forms of renal dropsy ; and further, that not every case of renal anasarca is necessarily an object for the systematic deprivation of chloride. It is in cases especially of acute and chronic parenchymatous nephritis that the question of chloride deprivation occurs, whilst the treatment of dropsy in contracted granular kidney falls into the same category as that of cardiac dropsy. In anasarca, due either to acute or chronic parenchymatous nephritis, this treatment is indicated. Cases of the latter in which elimination in general, and more especially that for sodium chloride, is deficient, can be recognised by two symptoms : (1) with an average intake in the food, the daily output, both per cent. and in total quantity in the urine, is extremely small ; and (2) running parallel with this deficient excretion, a progressive increase in body-weight, indicating a retention of sodium chloride in the tissues, and with it of water. Without visible anasarca a considerable quantity of water can thus be held back in the tissues. In this condition, then, and in anasarca itself, deprivation of chloride should be a part of the treatment, and is obtained by (1) regulating the intake in the food, and (2) increasing its elimination from the body by giving preparations of caffein, of which diuretin is the most effective, increasing both the total quantity of urine and also the percentage of chloride.

So also Minkowski, writing on the treatment of dropsy by regulating the intake of water and salt,[2] puts the reduction of sodium chloride intake in the first line in the treatment of renal dropsy. Limitation of fluid must be carried out with caution.

[1] *Folia Therap.*, 1907, i. 122.
[2] *Therap. d. Gegenw.*, 1907, n.f., ix. 1.

Excessive quantities of fluid are, however, certainly to be avoided, so that a diet of milk only—three litres of milk being taken daily —is a mistake, especially in the contracted kidney. One to one and a half litres of milk should be given combined with other articles of diet. It is well known that in renal dropsy, digitalis and the diuretics belonging to the group of purin bodies—caffein, diuretin, agurin, theocin—are often of great service. They bring about the elimination of a quantity of water and salt from the body without damaging the kidneys. The question cannot, however, be regarded as yet settled, for Drs. A. Bittorf and G. Jochmann, in an article on "Sodium Chloride Metabolism,"[1] state that the conditions present in renal disease are very variable. If cardiac failure is present, the conditions are the same as in uncompensated heart disease. The elimination of chloride is good, and to a great degree independent of the excretion of water. By increasing the amount of salt taken in acute nephritis with œdema there was brought about an increased output of sodium chloride and water, and afterwards the same patient, when without œdema and with good excretion of water, sometimes showed a delayed excretion of chloride. In chronic parenchymatous nephritis, there is very good, seldom delayed elimination of chloride with normal output of water. In chronic interstitial nephritis there is generally good chloride excretion.

J. Michell Clarke.

1 *Deutsches Arch. f. klin. Med.*, 1907, lxxxix. 485.

